# Enhanced Water Surface Object Detection with Dynamic Task-Aligned Sample Assignment and Attention Mechanisms

**DOI:** 10.3390/s24103104

**Published:** 2024-05-14

**Authors:** Liangtian Zhao, Shouqiang Qiu, Yuanming Chen

**Affiliations:** School of Civil Engineering and Transportation, South China University of Technology, Guangzhou 510641, China

**Keywords:** object detection, YOLOv8, sample assignment, unmanned surface vehicles, deep learning

## Abstract

The detection of objects on water surfaces is a pivotal technology for the perceptual systems of unmanned surface vehicles (USVs). This paper proposes a novel real-time target detection system designed to address the challenges posed by indistinct bottom boundaries and foggy imagery. Our method enhances the YOLOv8s model by incorporating the convolutional block attention module (CBAM) and a self-attention mechanism, examining their impact at various integration points. A dynamic sample assignment strategy was introduced to enhance the precision of our model and accelerate its convergence. To address the challenge of delineating bottom boundaries with clarity, our model employs a two-strategy approach: a threshold filter and a feedforward neural network (FFN) that provides targeted guidance for refining these boundaries. Our model demonstrated exceptional performance, achieving a mean average precision (mAP) of 47.1% on the water surface object dataset, which represents a 1.7% increase over the baseline YOLOv8 model. The dynamic sample assignment strategy contributes a 1.0% improvement on average precision at the intersection over union (IoU) threshold of 0.5 (AP^0.5^), while the FFN strategy fine-tunes the bottom boundaries and achieves an additional 0.8% improvement in average precision at IoU threshold of 0.75 (AP^0.75^). Furthermore, ablation studies have validated the versatility of our approach, confirming its potential for integration into various detection frameworks.

## 1. Introduction

As the global demand for resources continues to escalate, the industry for ocean resource development is experiencing robust growth, which has led to increasingly stringent performance requirements for oceanic equipment [1,2]. Unmanned surface vehicles (USVs), with their exceptional safety, efficiency, maneuverability, and strong adaptive capabilities, are increasingly becoming invaluable assistants in maritime operations [3]. Perception technology, a prerequisite component of USV operations, is instrumental in activities such as resource exploration and water purification [4]. Additionally, object detection algorithms hold extensive potential for application across various domains, including environmental monitoring, maritime traffic management, and water surface cleanup [5]. The integration and refinement of these algorithms are of significant importance to the promotion of sustainable development within the maritime economy [6]. While infrared thermal imagers may lack detailed imagery, and laser radars are prone to sea wave reflections, visible light cameras offer a cost-effective and robust solution for long-range object detection and have become the most common choice for USVs [7,8].

With the advancement of general object detection technology, models representing the one-stage object detection paradigm, such as YOLO [9], and those exemplifying the two-stage object detection approach, like R-CNN [10], have undergone optimization in their model structure and sample assignment methods. Consequently, there has been a substantial improvement in both accuracy and speed. However, object detection on water surfaces presents unique challenges, including the impact of sea fog and the difficulty in accurately delineating bottom bounding boxes, especially in densely packed scenes [11]. These challenges are further compounded by the limited computational resources and power supply of edge devices typically used for water surface object detection.

Current water surface object detection models predominantly focus on lightweight models and the detection of small targets [7,12,13,14], often neglecting the unique characteristics of water surface objects, such as the submerged nature of their bottom boundaries, as well as the distinctive attributes of water surface imagery, which typically features pronounced separation between the foreground and background. This oversight may limit the effectiveness of these models in accurately detecting and classifying objects within the aquatic environment. To tackle these challenges, this paper introduces an innovative water surface object detection method based on the YOLOv8 [15] framework. The contributions are summarized as follows:

- This paper introduces a threshold filtering strategy and a feedforward neural network (FFN) to address the issue of unclear bottom bounding boxes. The threshold strategy filters out the upper peak corresponding to the water surface boundary line in bimodal phenomena, while the FFN refines the prediction of the bottom bounding box position with negligible computational overhead.
- This paper proposes a novel label assignment strategy that synergizes the simplified optimal transport assignment (simOTA) [16] algorithm with task alignment learning (TAL) [17]. This strategy dynamically assigns labels, aligns localization loss with classification loss, and overcomes the limitations of fixed positive sample numbers in TAL, allowing for a more adaptive approach to label assignment, thereby improving the overall detection performance.
- This paper enhances the YOLOv8 backbone with the attention mechanism to improve modeling capabilities across the entire image and explore the combined application of a defog algorithm. Specifically, this paper introduces a C2f-att module and strategically incorporates the CBAM and transformer modules at various points within the network. This approach aims to enhance the model’s feature extraction capabilities and improve its responsiveness to critical visual cues. Our experiments on a water surface object detection dataset demonstrate the effectiveness of this approach when compared to the original YOLOv8 algorithm.

## 2. Related Work

### 2.1. Object Detection Models

Object detection models are primarily classified into two broad categories based on their feature extraction techniques: convolutional neural network (CNN)-based and transformer-based models. Transformer-based models, such as Co-DETR [18], DINO [19], and BEiT [20], harness the power of self-attention modules to extract information. This approach enhances the context comprehension ability, leading to improved performance. However, the computational costs of self-attention mechanisms can render these models less efficient when deployed on edge devices with limited resources.

On the other hand, CNN-based models can be further divided into one-stage and two-stage models. The two-stage models, as exemplified by the R-CNN [10] series, involve an initial selection of regions of interest (ROIs) from images, followed by a classification stage where a classifier refines object classification based on the region proposal. Fast R-CNN [21] and Faster R-CNN [22] refine the R-CNN architecture, integrating CNNs into both the classifier and region proposal stages to create an end-to-end detection model. While this explicit region proposal stage enhances orientation accuracy, it can also result in slower computation times.

The YOLO [9] series represents the one-stage object detection paradigm, which abandons explicit region extraction and predicts objects directly. Building upon the foundation of YOLOv1, YOLOv2 [23] introduces batch normalization to bolster the model’s robustness and employs anchors to refine accuracy. YOLOv3 [24] utilizes the feature pyramid network [25] (FPN) for multi-scale object detection, while YOLOv5 [26] employs adaptive anchor calculations and mosaic data enhancement to further enhance performance. Subsequent iterations like YOLOv6 [27], YOLOv7 [28], YOLOv8, and YOLOv9 [29] refine the backbone modules, reengineer the gradient propagation paths, and enhance model convergence. These models adopt an anchor-free approach, liberating them from the constraints of anchor boxes and enabling more flexible object matching across various shapes. Additionally, they employ a decoupled detection head to distinguish between classification and detection tasks, streamlining the overall detection process.

For water surface object detection tasks, the constrained computational resources and the necessity for real-time image processing demand a reduction in computational expense. In this study, we selected the YOLOv8 model as our foundational architecture and implemented significant enhancements to optimize its efficiency and accuracy.

### 2.2. Sample Assignment

Sample assignment in object detection is predominantly grounded in the intersection over union (IoU) metric. Early YOLO iterations employed a straightforward max-IoU matching strategy, where each ground truth was matched to a single positive sample. To mitigate the imbalance between foreground and background classes, subsequent models have augmented the number of positive samples through techniques such as multi-anchor, cross-grid, cross-branch, and cross-anchor predictions.

Zheng Ge et al. [30] introduced a novel perspective by framing sample assignment as an optimal transport (OT) problem, proposing the optimal transport assignment (OTA) method for object detection. This dynamic strategy not only adjusts the number of positive samples but also expedites model convergence. Building on this, YOLOX [16] introduced a simplified version of OTA, known as SimOTA, to further reduce computational overhead.

Chengjian Feng et al. [17] addressed the misalignment between classification and localization losses with TAL. By devising a new loss function, TAL aligns these two losses, enhancing overall model performance. Li Xiang [31] tackled the inconsistency in quality estimation and classification between training and inference with the generalized focal loss (GFL). Departing from the Dirac delta distribution, GFL employs a vector representation for box locations, enhancing prediction flexibility. This approach led to the discovery of a correlation between bounding box distribution statistics and actual localization quality, culminating in the development of the distribution-guided quality predictor (DGQP) in GFLv2 [32].

The recent YOLO series works have all adopted TAL for sample assignment. However, they selected a fixed number of positive samples and were unable to adjust them according to the quality of prediction and the number of targets. In some dense scenarios (such as multiple ships occluding each other), it failed to respond adequately, leading to potential missed detections or a decrease in detection accuracy, thereby affecting the operation of USVs. This paper introduces an enhanced version of the TAL algorithm that dynamically determines the number of positive samples based on the quality of the predictions, thereby improving the overall accuracy and efficiency of the object detection process.

### 2.3. Attention Mechanism

The attention mechanism plays a pivotal role in enhancing the representational capabilities of object detection models by selectively emphasizing or suppressing features. This approach is extensively integrated across various models. Traditional attention mechanisms are categorized into channel attention, spatial attention, branch attention, and hybrid attention [33].

The spatial transformer network [34] (STN) pioneers the localization net for identifying RoIs within an image. Squeeze-and-excitation networks (SENet) [35] incorporate a squeeze-and-excitation (SE) block to capture global information, while ECA-Net [36] innovates by substituting fully connected layers with 1D convolutions and introduces a channel attention (ECA) block, effectively reducing computational costs. Selective kernel networks (SKNet) [37] employs split, fuse, and select operations to adaptively adjust receptive fields (RFs).

Park et al. [38] introduced the bottleneck attention module (BAM), which parallelly infers spatial and channel attention for an input using 1 × 1 convolutions and global average pooling (GAP). The attention maps are then broadcasted to the same dimension for summation. At the same time, Woo et al. [39] proposed the convolutional block attention module (CBAM), which sequentially stacks spatial and channel attention blocks, utilizing GAP and global max pooling (GMP) to aggregate global information for each attention block.

Furthermore, the introduction of the transformer module has led to state-of-the-art (SOTA) achievements in numerous deep learning tasks. In the realm of object detection, models like Swin-transformer [40] and DETR [41] adopt self-attention to extract features and collect global information. Nonetheless, the substantial computational costs of the transformer module limit its application on edge devices with constrained resources.

Traditional attention mechanisms have been recognized for enhancing model accuracy with minimal increases in computational cost, whereas self-attention blocks, while offering superior performance, incur a significant computational overhead. This paper proposes an innovative integration of the CBAM and a self-attention block into the network backbone. To mitigate the computational expense associated with the self-attention block, we replaced it within the bottleneck layers, optimizing the model by reducing the number of channels. This approach effectively balances the trade-off between performance enhancement and computational efficiency.

### 2.4. Water Surface Object Detection

Within the domain of water surface object detection, contemporary research has been predominantly concentrated on the refinement of detection algorithms for small targets and the engineering of lightweight model architectures. Li et al. [42] introduced the DENS-YOLOv6 model, tailored for the detection of aquatic debris, which incorporates an adaptive noise suppression module to curtail the disruptive effects of noise on the detection of diminutive objects at the water’s surface. Zhang et al. [13] leveraged reparameterization techniques for feature extraction and propose a spatial to depth convolution strategy to enhance the detection of small targets on water surfaces. Chen et al. [43] augmented the capabilities of YOLOv5 for the detection of minor floating objects on water surfaces through the application of SIoU and soft NMS. In parallel, another study by Zhang et al. [12] integrated multi-level features to refine the faster R-CNN model, thereby amplifying the velocity of water surface detection tasks. Li et al. [44] introduced enhancements to the SSD algorithm, effectively addressing the challenge of non-detection zones that commonly plague water surface target detection scenarios. Ma et al. [45] present an innovative approach by integrating CLIP into the realm of water surface garbage object detection, advocating a method that synergizes both strong and weak supervisory signals. Dong et al. [46] introduced Marit-YOLO, a model underpinned by multi-scale pyramid attention networks, designed to expedite the process of feature fusion within the detection pipeline. Chen et al. [47] devised a water surface garbage detection model predicated on the lightweight YOLOv5 framework, effectively reducing the parameter count to facilitate the model’s deployment on edge computing devices. Furthermore, Dong et al. [48] proposed a lightweight convolutional architecture integrated with a fused attention module, which not only diminishes the model’s parameter load but also accelerates computational throughput. Addressing the challenge of few-shot learning scenarios, Wang et al. [14] introduced an adaptive lightweight small object detection methodology predicated on High-Resolution-Net, delivering contributions of significance to the dual objectives of model lightweighting and detection accuracy.

These works reflect the ongoing efforts within the academic community to refine object detection models specifically for the challenges presented by water surface detection tasks, such as the presence of small, distant, or partially obscured objects. However, these enhancements have predominantly focused on methodologies for feature extraction and model lightweighting, with a relative dearth of targeted adjustments tailored to the unique characteristics of water surface targets. Additionally, there is a scarcity of models where the SOTA networks are being leveraged for the purpose of water surface object detection.

## 3. Proposed Approach

### 3.1. Dynamic Sample Assignment Method

This paper introduces a novel strategy designed to achieve dynamic label assignment, effectively aligning localization loss with classification loss in object detection tasks. This strategy is underpinned by two critical components of loss computation: classification loss and localization loss.

In scenarios where an anchor has a high IoU value but a low classification score, it can negatively impact the model, potentially suppressing the predictions of correctly classified anchors. To rectify this misalignment, we introduce a task-aligned loss function, expressed as

(1) $$t=s^{\alpha }\times u^{\beta },$$

where s represent the classification score, u denotes the IoU value, α and β are used to modulate the influence of classification and localization tasks. The goal of this task-aligned loss t is to balance and jointly optimize both tasks.

For evaluating classification quality, a binary cross entropy (BCE) loss was employed. To facilitate model convergence, we normalized t and introduced a new term $\hat{t}$ with Formula (2), which replaces the original task-aligned loss in our training process:

(2) $$\hat{t}=\frac{t-\mu }{\sigma },$$

where μ represents the expected value and σ represents the variance. Additionally, focal loss [49] was incorporated as Formula (3) to mitigate the imbalance between positive and negative samples.

(3) $$L_{cls}=\sum_{i-1}^{N_{pos}}|{\hat{t}}_{i}-s_{i}{|}^{\gamma }BCE(s_{i},{\hat{t}}_{i})+\sum_{j=1}^{N_{neg}}s_{j}BCE(S_{j},0)$$

where γ represents the focusing parameter.

For localization loss, we utilized the center-weighted intersection over union [50] (CIoU) metric as

(4) $$L_{reg}=\sum_{i=1}^{N_{pos}}{\hat{t}}_{i}L_{CIOU}(b_{i},{\tilde{b}}_{i}),$$

where $b_{i}$ and ${\tilde{b}}_{i}$ represent the predicted bounding boxes and the corresponding ground truth boxes, respectively.

Instead of selecting a fixed number of positive samples, our strategy dynamically adjusts this number based on prediction quality. The settled top m anchors with the highest IoU values are chosen to represent the quality of predictions and are used to determine the dynamic k parameter as the number of positive samples. This approach does not incur additional computational costs, as the IoU values are pre-calculated and incorporated into the loss function.

Finally, the top k predictions with the lowest $\hat{t}$ values were selected as positive samples. This dynamic sample assignment strategy not only enhances prediction accuracy but also expedites the convergence of the model, leading to more efficient training and improved performance.

### 3.2. Unclear Bottom Boundaries Prediction

Water surface objects, such as those found in seas or lakes, present unique challenges for detection models due to their floating nature and the potential submersion of their bottom parts, leading to ambiguous bottom boundaries. Conventional detection models often misidentify the bottom boundary, either as the interface between the object and the water surface or as an arbitrary position within the water. To address this issue, this paper proposes two strategies.

**Figure 1:**
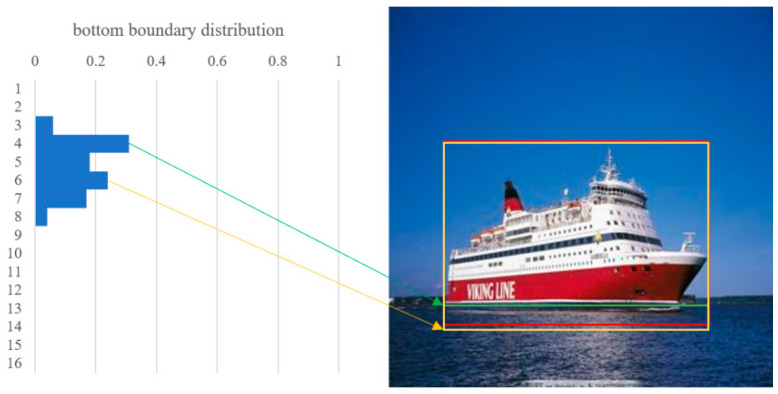
The prediction of an image. The ground truth is denoted by a red bounding box. We identified and reflected two distinct peaks in the bottom boundary distribution, represented by the green and yellow bounding boxes. The green box corresponds to the boundary that is more inclined towards the interface between the object and the water surface. In contrast, the yellow box is positioned closer to the actual bottom of the object, providing a more accurate representation of the object’s submerged portion.

The first strategy leverages threshold filtering. Similar to the GFL approach, this paper employs a prediction head capable of estimating arbitrary distributions. As Figure 1 shows, our analysis reveals a distinct bimodal pattern in the majority of images, with two prominent peaks in the probability distribution. The upper peak tends to predict the interface between the object and the water, while the lower peak is more likely to represent the true bottom boundary of the object. 

From this perspective, this paper manually chose the lower peak as the bottom boundary, which can be expressed as

(5) max(Pick(D,2)),

where D represent the values from distribution, and Pick(·,2) means pick the two peaks with the largest values.

In practice, a manually chosen tends to neglect other parts of information in the probability distribution. To refine this manual selection process, this paper introduces a learnable parameter γ to balance the influence of the peaks and the overall probability distribution. The bottom boundary is then calculated using the following formula:

(6) $$O=\gamma v_{p}+(1-\gamma )v_{d}$$

where $v_{p}$ represents the value predicted by the lower peak, and $v_{d}$ represents the value from the general distribution.

The second strategy involves an additional FFN that follows the probability distribution prediction. This FFN, equipped with a single hidden layer, takes the 16 prediction probabilities as input and refines the regression of the bottom boundary. It can be expressed as

(7) $$y=\phi (W_{2}(\phi (W_{1}x+b_{1}))+b_{2}),$$

where W is the weight matrix containing all the weight vectors, b is the vector containing all the bias terms, and ϕ is the sigmoid activation function.

Despite its minimal input and straightforward architecture, this FFN contributes to a 0.5% increase in mean average precision (mAP) while only adding a modest computational load of a few hundred floating point operations (FLOPs). This approach demonstrates our commitment to enhancing detection accuracy without significantly increasing computational costs.

### 3.3. Attention Mechanism Integration

**Figure 2:**
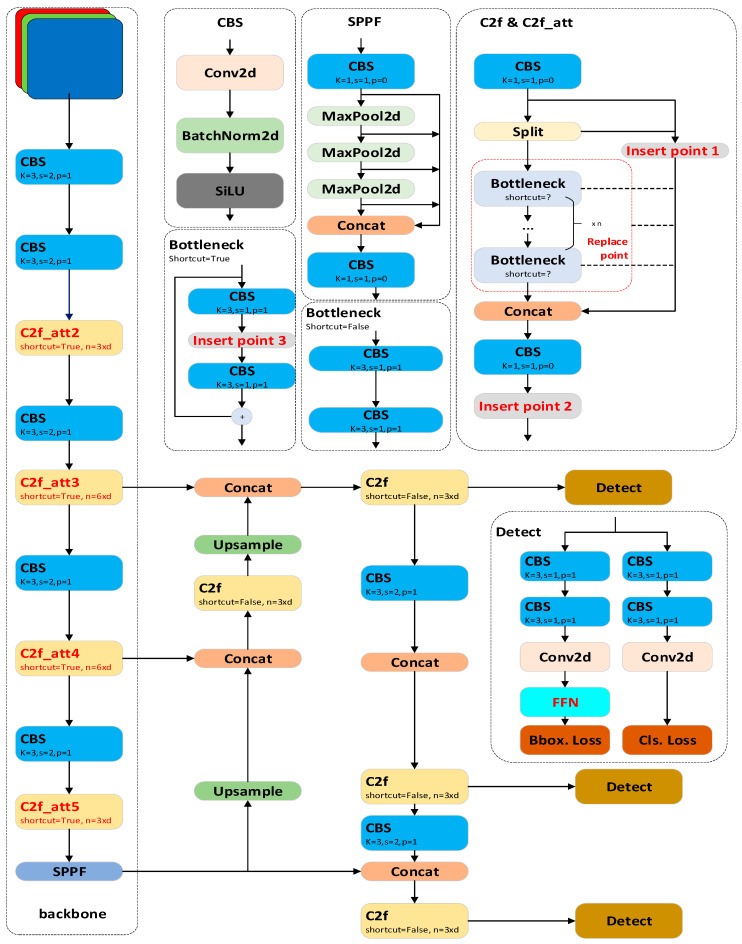
The overall structure of the proposed model.

In the design of our model, as Figure 2 shows, we adhered to the same architectural paradigm as the YOLOv8 model to control for variables. We elected to utilize the sigmoid-like unit (SiLU) as the activation function, which is concatenated with the 2D convolution (Conv2d) and 2D batch normalization (BatchNorm2d) to form the CBS module. The model is segregated into three distinct components: the backbone, the neck, and the detection head. Within the backbone, we implemented the fast version of the cross-stage partial (CSP) bottleneck with 2 convolution (C2f) modules and spatial pyramid pooling—fast (SPPF) modules to expedite the feature extraction process. For the neck design, we adopted a structure that integrates the path aggregation network (PAN) with the FPN to amalgamate information from different feature maps. Ultimately, we employed a decoupled detection head that independently learns to correct localization and classification errors.

Drawing upon the YOLOv8 model as our foundation, this paper incorporates attention mechanisms into our model to enhance its feature extraction capabilities. Our exploration includes the CBAM and the self-attention module. To achieve this, this paper developed a C2f with attention mechanism (C2f-att) module and identifies three insert points and one replace point within the model architecture for the application of these attention modules. In the C2f and C2f_att module, when the attention mechanism is not applied, the connections involved in insert points are identity. When the attention mechanism is applied, it will be placed in insert points or be used to replace the bottleneck module. Besides adding the attention mechanism after the C2f module (insert point 2), we also applied the attention mechanism in insert point 3 and the replace point. These integration points, which involve fewer channels, are instrumental in reducing computational complexity and accelerating the inference process.

The parameters of the self-attention module are defined as follows:

(8) $$P=l(12h^{2}+13h)+Vh,$$

where l represents the number of layers, h denotes the dimension of the hidden layers, and V is the number of bag-of-words (BOW). The FLOPs can be approximated as

(9) 6×T×P,

where T is the token size. The output of the self-attention module retains the same dimension as the input. Thus theoretically, the self-attention module can be integrated at any point. For the substantial computational costs brings by token count and hidden layer dimensions, this paper chose to incorporate the self-attention module solely in the C2f_att5, which is the minimum feature map. This paper also experimented with different patch sizes and stacking configurations to identify the optimal model structure.

In addition to the self-attention module, this paper incorporated the CBAM into our model. The CBAM sequentially applies channel and spatial attention, utilizing GMP and GAP across the feature map’s channel and spatial dimensions. For a selected feature map F, the channel attention can be expressed as

(10) $$M_{c}(F)=\phi (\mathrm{MLP}(\mathrm{AvgPool}(F))+MLP(\mathrm{MaxPool}(F))),$$

and the spatial attention can be expressed as

(11) $$M_{s}(F)=\phi \left(f^{7\times 7}([\mathrm{AvgPool}(F);\mathrm{MaxPool}(F)])\right)=\phi \left(f^{7\times 7}\left(\left[F_{\mathrm{avg}\;}^{s};F_{\max\;}^{s}\right]\right)\right),$$

where $f^{7\times 7}$ represents a 7 × 7 convolution.

The resulting attention weights are then fused with the original input via a fully connected (FC) layer or a CNN layer to produce an attention-weighted feature map. The CBAM minimally impacts the model’s parameter count and FLOPs, and like the self-attention module, it maintains the input shape and can be flexibly placed within the model. This study applied the CBAM module at each of the designated integration points.

## 4. Experiments

### 4.1. Dataset

We employed an open-source water surface object detection dataset that contributes to the Paddle AI community. The original baseline model utilizing YOLOv5 achieved a mAP of 30.8%. Subsequently, we conducted preliminary testing with the YOLOv8 framework, which yielded a notably enhanced mAP of 45.4%. We adopted this latter performance as the benchmark for our current research endeavors.

The dataset comprises ten categories of water surface targets that are representative of typical scenarios encountered by USVs. These categories include the lighthouse, sailboat, buoy, rail bar, cargo ship, naval vessels, cruise liner, dock, submarine, and fishing boat. The dataset is designed to simulate a comprehensive range of real-world conditions, featuring these targets under various weather conditions and from multiple shooting angles, thereby encapsulating the diverse environments in which USVs operate.

To facilitate a balanced training and evaluation process, we randomly partitioned the dataset into a training set and a test set, maintaining a 3:7 ratio. This division resulted in a training dataset comprising 4015 images and a test dataset with 1721 images. Additionally, there were partly images including instances where objects were obscured from one another. This feature enhances the dataset’s realism and challenges the detection model’s ability to identify and classify targets even under occlusion.

### 4.2. Experimental Setup

To substantiate our proposed strategy and investigate the impact of the attention mechanism, a series of experiments were carried out. Utilizing the Torch library and the OpenMMLab codebase, we trained our model on an NVIDIA GTX 3070ti GPU, leveraging the CUDA 11.2 GPU acceleration library for enhanced performance. To ensure a fair comparison, we selected the YOLOv8s model as our baseline, maintaining consistent model scales.

We set the batch size to 4 and trained 300 epochs with a 0.01 learning rate. The first 100 iterations were set as a warm-up period and the weight decay was set to 0.0005. To underscore the effectiveness of our approach, we mirrored the training configurations and data preprocessing steps of the YOLOv8s model as implemented in OpenMMLab.

The model’s performance was assessed across three key dimensions: prediction accuracy, computational cost, and model size. We employed AP^threshold^ and mAP^0.5:0.95^ as metrics for prediction performance. AP^threshold^ measures the precision for a predefined IoU threshold, while mAP^0.5:0.95^ provides a comprehensive assessment of the model’s ability to detect objects at various IoU levels. We used the FLOPs to evaluate the computational costs and used the parameter count to evaluate the model size. These evaluations ensure a balanced perspective on the trade-offs between accuracy, efficiency, and complexity in our detection model.

### 4.3. Experiment on Dynamic Sample Assignment

**Figure 3:**
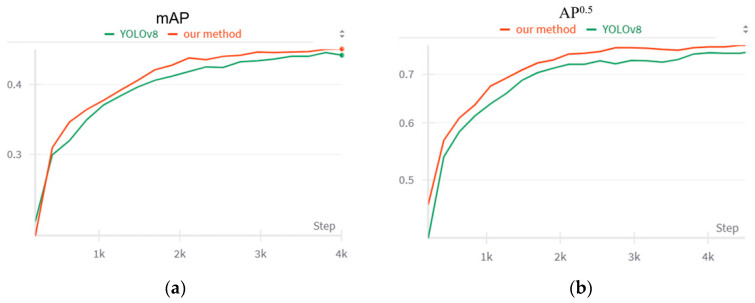
Comparison of prediction accuracy and convergence speed between our method and the YOLOv8 model. (**a**) Comparison of mAP between our method and the YOLOv8 model. (**b**) Comparison of AP^0.5^ between our method and the YOLOv8 model.

We carried out experiments to illustrate the advantages of our dynamic assignment strategy. Figure 3 shows the learning curve of our method and the original YOLOv8s model.

The results of our experiments clearly demonstrate that our model not only excels in prediction performance but also achieves faster convergence compared to the YOLOv8 model. Our strategy achieved a mAP of 44.6 in just 2100 training steps, a milestone that the YOLOv8 model requires 3300 steps to reach. This represents a significant acceleration in the training process.

Furthermore, our model shows a notable improvement in mAP by 0.8% and a more pronounced enhancement in the AP^0.5^ by 1.0% and in the mAP on large objects by 1.1%. These enhancements underscore the effectiveness of our dynamic assignment strategy in refining the detection accuracy and the overall robustness of the model in object detection tasks.

**Figure 4:**
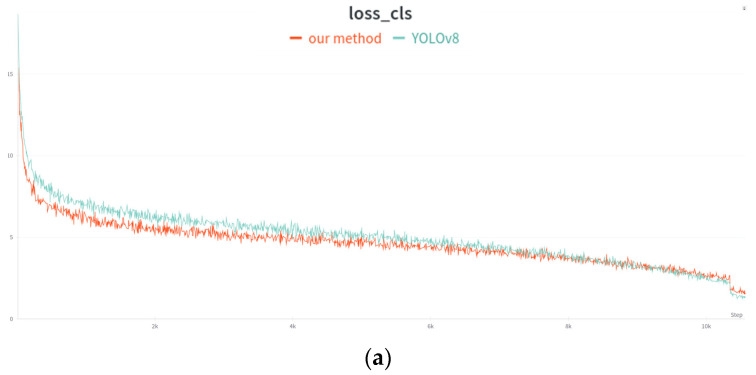

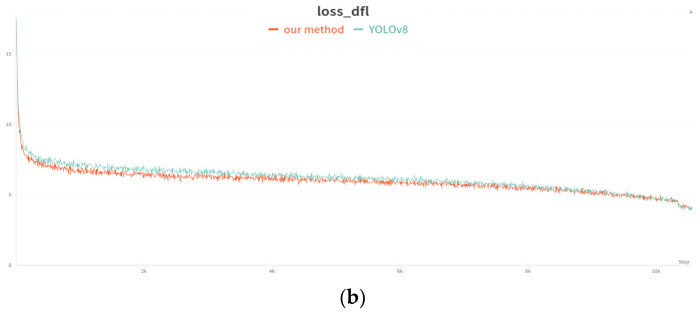
Comparison of classification loss and DFL loss between our method and the YOLOv8 model. (**a**) Comparison of classification loss between our method and the YOLOv8 model. (**b**) Comparison of DFL loss between our method and the YOLOv8 model.

Figure 4 illustrates the comparative analysis between our proposed method and the original YOLOv8 model concerning classification loss and distribution focal loss (DFL). It is evident that the implementation of our novel strategy for dynamic task alignment in matching positive and negative samples leads to a reduction in both aforementioned losses, thereby achieving enhanced model performance.

### 4.4. Experiment on Bottom Boundary Prediction

Within the dataset, we encountered scenarios where objects were partially submerged or appeared small and shallow in the water. This resulted in the bottom boundary of the object being close to the boundary between the object and the water surface, making it challenging to distinguish in our observations. To address this, we defined the bimodal phenomenon based solely on the distinct values of the two highest peaks in the probability distribution rather than regarding the distance between them. This approach allowed us to identify and differentiate the object’s submerged boundary more accurately. To refine our detection strategy, multiple thresholds were established for selecting the lower peak. The performance metrics associated with these thresholds are detailed in Table 1.

The threshold set to 0 corresponded to the baseline performance of the original YOLOv8 model. As indicated in Table 1, the occurrence of the bimodal phenomenon, irrespective of the distance between peaks, was observed in 35.5% to 84.4% of the objects, which increased by 28.7% to 73.6% compared to the definition, considering the distance.

At a threshold of 0.1, we observed a marginal improvement in the mAP by 0.2% and a more significant enhancement in the AP^0.75^ by 0.4%. The manually chosen boundary tended to ignore the general probability distribution information that could lead to a bad impact. By introducing a balance parameter that merged information from both peak values and general probability distribution, our model achieved a 0.3% increase in mAP.

Notably, as the threshold increased, there was a slight drop in model performance by 0.2%. This decrement may have been due to a more lenient threshold that incorrectly classifies some distinct boundaries. These findings underscore the importance of fine-tuning the threshold to strike the right balance between sensitivity and specificity in object detection tasks.

Our FFN strategy involves varying the number of hidden layers and the dimensions of these layers to optimize performance. The experimental outcomes, as presented in Table 2, indicate that an FFN configuration with a single hidden layer and four channels within that layer yielded a 0.5% enhancement in mAP and a 0.8% increase in the AP^0.75^. However, when employing a deeper network with higher-dimensional layers, we observed a slight decrease in performance, potentially due to overfitting.

Both approaches demonstrated significant improvements in the AP^0.75^, which is attributed to the models’ increased focus on refining the prediction of the bottom boundary. Despite the additional parameters, we advocate for the FFN strategy, as it delivers better performance on the dataset and benefits from hardware acceleration.

**Figure 5:**
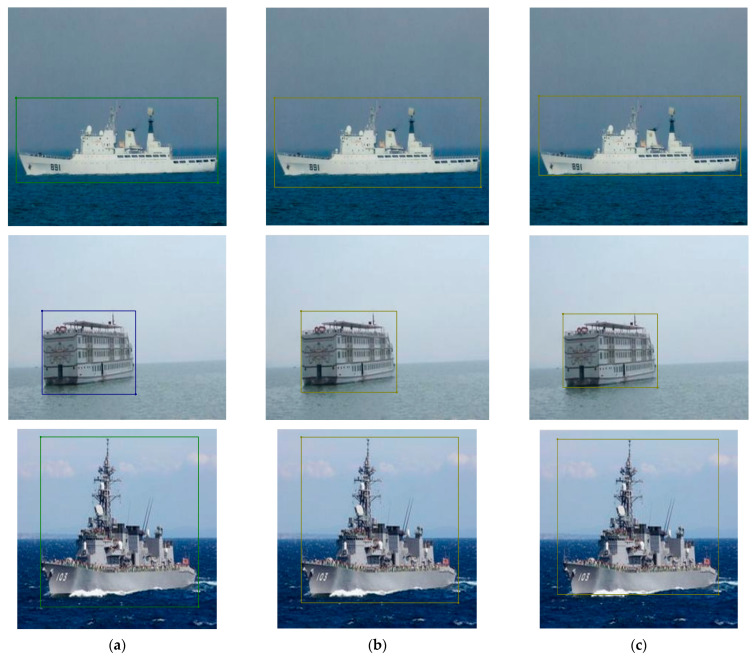
Comparison of bottom boundary prediction between the model developed in this study and the YOLOv8s model. (**a**) the actual boundary box of the target (**b**) the predictive output from the model developed in this study (**c**) the predictive output from the original YOLOv8 model.

Figure 5 presents a comparative analysis of the bottom boundary predictions for surface objects between the model developed in this study and the original YOLOv8 model. In this figure, (a) signifies the actual boundary box of the target, (b) represents the predictive output from the model developed in this study, and (c) indicates the predictive output from the original YOLOv8 model. The output from our proposed strategy accounts for the size and orientation of the surface target to predict the position of the bottom boundary, in contrast to the YOLOv8 model, which has the propensity to predict directly at the point where the target intersects with the water surface.

Additionally, we computed the bottom boundary loss, as defined by Formula (12), to substantiate the superiority of the method proposed in this paper.

(12) $$L_{BB}=\sqrt{\left|q_{i}-{\bar{y}}_{i}\right|}$$

where $q_{i}$ denotes the position of the i-th predicted bottom boundary, while ${\bar{y}}_{i}$ represents the true value of the i-th predicted bottom boundary. In the design of the bottom boundary loss, we eschewed the conventional L1 loss in favor of the square root function. This strategic choice was made to mitigate the imbalance that can arise from variations in the size of the target bounding boxes. Table 3 presents a comparative analysis of the bottom boundary loss between the model proposed in this study and traditional algorithms. It is clear to see that the incorporation of the bottom boundary prediction strategy developed in this research can effectively guide the model to predict the submerged lower boundaries more accurately.

### 4.5. Experiment on Attention Mechanism

This paper examined the influence of patch size and hidden layer hyperparameters, including dimensions and layers, within the self-attention module. To save computational resources, we limited the FLOPs addition to 2.8 GFLOPs. The result is shown in Table 4. Our results indicate that a 2 × 2 patch size, paired with a hidden layer of 512 dimensions, successfully improved mAP by 0.3%, and this will be utilized in the subsequent experiments.

Our approach involves the strategic application of the attention mechanism at three insert points and one replace point within the model’s architecture. Our analysis not only scrutinized the individual impacts of these placements but also explored the combined effects of integrating both self-attention and CBAM in various sections of the backbone. The outcomes of these experiments are compiled in Table 5, Table 6 and Table 7, providing a comprehensive view of how the synergistic use of these attention modules contributes to the overall performance of the model.

Our experiments revealed that both attention mechanisms experienced a minor decrease in mAP when applied at insert point 1, which suggests that the residual connections within a deep neural network played a significant role. When the self-attention module was employed at the replace point, it operated with half the number of channels and yet managed to achieve a similar mAP increase as when applied at insert point 2.

The synergistic application of the CBAM and the self-attention module yielded a higher mAP compared to using CBAM alone. This improvement was likely due to the model learning distinct attention patterns that enhance its feature representation capabilities. However, this combined approach came at the cost of an 8.8% increase in computational cost and a 22% increase in the number of parameters. We recommend using the self-attention block only with adequate computing power. By carefully orchestrating the placement and combination of attention mechanisms, this study aimed to enhance the model’s feature extraction capabilities and improve its detection accuracy.

### 4.6. Ablation Experiments

To evaluate the impact of various enhancements on our model, this paper conducted a series of ablation experiments. These experiments aimed to isolate and measure the contributions of the following improvements: the CLAHE defog algorithm, dynamic sample assignment strategy, bottom boundary prediction strategy, CBAM, and the self-attention module. The outcomes of these experiments are systematically presented in Table 8.

The CLAHE algorithm enhances image contrast, which is particularly beneficial for perceiving objects in low-light conditions. When integrated with the attention mechanism, CLAHE can contribute to a more robust prediction performance. However, for images free of haze, we typically deactivate CLAHE as its application increases processing time by 14%.

Our experiments show that the dynamic sample assignment, bottom boundary prediction strategy, and attention mechanism had minimal influence on each other, indicating their potential for independent optimization. Individually, dynamic sample assignment and bottom boundary prediction strategies improved the mAP by 0.8% and 0.5%, respectively. When these strategies were employed together, the mAP was further enhanced by 1.2%. This synergistic effect demonstrates the generalizability of these improvements, suggesting that they can be effectively applied to a variety of other models to enhance their object detection capabilities.

### 4.7. Comparison with Other Algorithms

To substantiate the superiority of the model proposed in this study, a comparative analysis was conducted with prevailing models, including Faster R-CNN (R-101-FPN), Cascade R-CNN (R-101-FPN), YOLOv5s (P5), YOLOv8s, and YOLOv9s, on a common dataset. Both mAP values and parameter count were computed for each model. The findings are delineated in Table 9.

In the absence of a self-attention module, our model achieved a 1.6% improvement in mAP over the YOLOv8 baseline model without a substantial increase in parameter count, as well as a 1.5% enhancement over the SOTA network, YOLOv9. The incorporation of a self-attention module further augmented the mAP by an additional 0.1%. These comparative experimental results underscore the superior performance of the model introduced in this research in the detection of water surface targets, effectively balancing the trade-off between predictive accuracy and speed.

## 5. Discussions

In the current landscape of water surface object detection algorithms, research has predominantly focused on enhancements aimed at lightweight models and the detection of small targets, with scant attention dedicated to the unique characteristics inherent to water surface targets. Considering the distinctive attributes of water surface imagery, this study introduces a novel water surface object detection system, significantly advancing the capabilities of USVs for maritime perception.

Our approach, which integrates the CBAM and a self-attention mechanism into the YOLOv8s model, endows the model an enhanced capability to focus on the foreground objects and has demonstrated substantial improvements in detection precision and computational efficiency. The use of the self-attention module, particularly in the most compressed feature maps, minimizes computational overhead while retaining performance gains. This paper also implemented the CLAHE algorithm to improve image contrast, particularly in low-light conditions, and enhance its capabilities for foggy image processing.

The dynamic sample assignment strategy effectively aligns localization and classification losses, allowing for a more adaptive selection of positive samples and the enhancing of the detection of large objects. This strategy not only enhances the efficiency for detecting large objects, increasing the mAP by 1.1%, but also saves about 36% of the iteration count when converging to the same accuracy.

The challenge of indeterminate bottom boundaries, common in water surface object detection, is addressed through our proposed threshold filtering strategy and FFN strategy. These methods provide targeted guidance for refining bottom boundaries, achieving a notable 0.8% improvement in AP^0.75^ with minimal additional computational overhead.

Ablation experiments conducted within this study validate the individual and synergistic contributions of the dynamic sample assignment, bottom boundary prediction strategies, and attention mechanisms to the overall performance. The generalizability of these improvements suggests their potential for integration into various detection frameworks, enhancing object detection capabilities across the board.

While our model demonstrated robust performance for water surface object detection, there are areas for future work. The computational costs associated with the self-attention mechanism, although minimized, could be further optimized for even more resource-constrained environments. In addressing the issue of occlusion caused by splashing waves, the approach adopted in this paper involves the use of cutout data augmentation in the lower half of the image. It could consider applying generative algorithms for image restoration and integrating such algorithms into the existing model framework in the future. Additionally, the generalizability of our model to other detection tasks and datasets warrants further investigation. Despite these considerations, our study presents a significant advancement in the field of object detection, particularly for USV applications, and it provides a solid foundation for future research and development in this domain.

## 6. Conclusions

This research presents a robust water surface object detection system that significantly outperforms the original YOLOv8s model, achieving a 47.1% mAP on the water surface object dataset. The dynamic sample assignment strategy, combined with the threshold-based and FFN approaches for bottom boundary prediction, as well as the integration of CBAM and self-attention mechanisms, led to a 1.7% improvement in mAP with negligible computational costs. Our model’s balance of high prediction accuracy with computational efficiency makes it a versatile solution for a wide array of water surface object detection tasks. The enhancements demonstrated in this study are not only confined to our model but also have the potential to be generalized and applied to other detection frameworks, offering a promising direction for future research and development in the field of water surface object detection.

## Figures and Tables

**Table 1:** Performance comparison with different thresholds and γ strategy.

Threshold	With Distance	Bimodal Phenomenon	AP^0.75^	mAP
0 (YOLOv8)		0%	52.0	45.4
0.05	√	6.8%	52.1	45.4
	×	35.5%	52.4	45.6
0.1	√	9.1%	52.0	45.3
	×	68.7%	52.4	45.6
0.15	√	10.8%	51.8	45.3
	×	84.4%	52.1	45.5
0.1 + γ	×	68.7%	52.6	45.7

**Table 2:** Analysis of different choices of hidden layers and hidden layer dimensions on the dataset.

Hidden Layers	Hidden Layer Dimensions	AP^0.75^	mAP
0		52.4	45.6
1	4	52.8	45.9
2	4 + 4	52.7	45.7
	8 + 4	52.7	45.7
3	8 + 4 + 4	52.3	45.4

**Table 3:** Comparison of bottom boundary loss between different models.

Models	L_BB_
Faster R-CNN	4.932
YOLOv8s	5.138
Our method	3.560

**Table 4:** Comparison between different patch sizes and hidden layer hypermeters.

Patch Size	Hidden Layers	Hidden Layer Dimensions	GFLOPs	Parameters	mAP
1 × 1	1	256	2.20	0.92 M	45.5
2 × 2	1	512	2.51	4.19 M	45.7
2 × 2	4	256	2.20	3.67 M	45.2
4 × 4	1	512	1.10	7.34 M	45.6

**Table 5:** Performance comparisons on different applied positions of CBAM.

C2f_att2&3&4&5	mAP
Insert point1	45.4
Insert point2	45.8
Insert point3	45.5
Replace point	45.6

**Table 6:** Performance comparisons on different applied positions of self-attention.

C2f_att5	mAP
Insert point1	45.3
Insert point2	45.7
Insert point3	45.6
Replace point	45.7

**Table 7:** Performance comparisons on utilizing CBAM and self-attention independently and synergistically.

Attention Mechanism	mAP
CBAM	45.8
self-attention	45.7
CBAM + self-attention	45.9

**Table 8:** Results of the ablation study.

CLAHE	Dynamic Sample Assignment	Bottom Boundary Prediction	CBAM + SA	mAP
×	×	×	×	45.4
√	×	×	×	45.1
×	√	×	×	46.2
×	×	√	×	45.9
×	×	×	√	45.9
×	√	√	×	46.6
√	×	×	√	46.1
×	×	√	√	46.4
×	√	×	√	46.6
×	√	√	√	47.0
√	√	√	√	47.1

**Table 9:** Comparison of mAP values across different models.

Models	mAP	GFLOPs
Faster R-CNN (R-101-FPN)	40.1	114.3
Cascade R-CNN (R-101-FPN)	44.0	131.1
YOLOv5s (P5)	39.8	16.5
YOLOv8s	45.4	28.6
YOLOv9s	45.5	26.4
Our model without self-attention	47.0	28.9
Our model with self-attention	47.1	31.3

## Data Availability

The original contributions presented in the study are included in the article, further inquiries can be directed to the corresponding author.

## Abbreviations

The following abbreviations are used in this paper:

USVs	unmanned surface vehicles
YOLO	you only look once
CBAM	convolutional block attention module
SA	self-attention
FFN	feedforward neural network
mAP	mean average precision
IoU	intersection over union
AP	average precision
SimOTA	simplified optimal transport assignment
TAL	task alignment learning
CNN	convolutional neural network
ROIs	regions of interest
FPN	feature pyramid network
GFL	generalized focal loss
GAP	global average pooling
GMP	global max pooling
BCE	binary cross entropy
CIOU	center-weighted intersection over union
FLOPS	floating point operations
CSP	cross stage partial
C2f	fast version of the CSP bottleneck with 2 convolutions
C2f-att	C2f with attention mechanism
